# Endothelin-1-induced hypertrophic alterations and heme oxygenase-1 expression in cardiomyoblasts are counteracted by beta estradiol: in vitro and in vivo studies

**DOI:** 10.1007/s00210-018-1462-z

**Published:** 2018-01-21

**Authors:** Tunde Barta, Agnes Tosaki, David Haines, Gyorgy Balla, Istvan Lekli, Arpad Tosaki

**Affiliations:** 1 Department of Pharmacology, Faculty of Pharmacy, University of Debrecen, Nagyerdei krt., 98, Debrecen, 4032 Hungary; 2 Department of Pediatrics, Medical and Health Science Center, University of Debrecen, Debrecen, Hungary; 3 Hemostasis, Thrombosis and Vascular Biology Research Group, Hungarian Academy of Sciences, Debrecen, Hungary

**Keywords:** Heart failure (HF), Endothelin-1 (ET-1), β-Estradiol (β-E), Heme oxygenase-1 (HO-1), Hypertrophy, H9c2 rat cardiomyoblast, In vitro, In vivo rat

## Abstract

Endothelin-1 (ET-1), a potent vasoconstrictor normally active in maintaining vascular tone, may mediate significant pathogenic effects, contributing to several serious diseases when aberrantly expressed or regulated. The present study evaluates the capacity of ET-1 to affect endothelin-1-associated hypertrophic activity and decreased expression of heme oxygenase-1 by H9c2 rat cardiomyoblasts in vitro, corresponding to in vivo processes underlying cardiovascular diseases (CVDs). Beta estradiol (β-E) is tested for its capacity to alter the effects of ET-1. H9c2 cells, cultured 48 h, were stimulated with 100–10,000 nM of ET-1 and evaluated for changes in cell size, cell viability, and expression of the cytoprotective heat shock protein heme oxygenase-1 (HO-1), with 200 nM of β-E included in selected cultures to evaluate its effect on ET-1-mediated changes. The application of 100 to 10,000 nM of ET-1 resulted in a significant increase in average cell size and decreases in both cell viability and HO-1 protein content (*p* < 0.05). Moreover, 200 nM of β-E was observed to significantly counteract these effects by cardiomyoblasts stimulated with 1000 nM of ET-1 (*p* < 0.05). Sprague-Dawley rats treated intravenously with 1000 ng/kg of ET-1 demonstrated reduced HO-1 expression in peripheral blood and left ventricular tissue, which was counteracted by injection of 200 ng/kg β-E—demonstrating a possible correspondence between in vitro and in vivo effects. An outcome of particular value for clinical use of β-E, in the management of cardiac hypertrophy, is the observed capacity of the drug to abate ET-1-mediated suppression of HO-1 expression. It has been previously demonstrated that HO-1 inducers exhibit potent cardioprotective properties, thus offering the promise of combining them with β-E, allowing lower effective dosage of the drug and concomitantly lower adverse side effects associated with its clinical use. Major findings of this investigation are that pretreatment of cardiomyoblasts with β-E inhibited their hypertrophic response to ET-1 and counteracts the decrease of cell viability. These effects were associated with a restoration of HO-1 protein expression in both under in vitro and in vivo conditions.

## Introduction

Cardiopulmonary and cardiovascular diseases (CVDs) leading to respiratory and heart failure are the major cause of death and incapacitation in the EU and in many other areas of the world, particularly in developed nations (Nichols et al. 2013). These syndromes prominently include abnormal increase in cardiomyocyte size, with resulting increase in heart size, acute myocardial infarction (AMI), ischemia-reperfusion (I/R) injury, and heart function deterioration following AMI (Nichols et al. 2013). Despite significant advances in early diagnosis, prevention and therapeutic interventions for these syndromes, CVD mortality continues to increase worldwide, a phenomenon that is particularly problematic among aging populations (Brutsaert 2010). Death rates among persons afflicted with CVD are very high. More than 50% of heart failure patients die within 1–4 years of diagnosis, and most afflicted persons experience significant debilitation during periods of illness (Stewart et al. 2001). Currently, CVD imposes an enormously adverse impact on quality of life, leading to the disruption of daily management and increasing dependence on caregivers (Albert et al. 2002). Moreover, despite intensive research efforts, no drugs have yet been developed that definitively prevent or remediate CVD at levels that allow full restoration of normal life for those afflicted. It is therefore imperative to develop low-cost preventive and therapeutic countermeasures for these syndromes, which incorporate both pharmacological and non-drug interventions.

An essential precondition for identification of novel countermeasures to CVD is development of in vitro cell culture and animal models of cardiac cellular function that allow rapid screening of drugs and non-drug inducers of cardioprotective adaptive responses. The present investigation examines the effects of endothelin-1 (ET-1), a major physiologic inducer of hypertrophic changes, on an in vitro model of H9c2 rat cardiomyoblasts, in experiments that evaluate the capacity of the drug beta-estradiol (β-E) to attenuate these effects. Pharmacological strategies for decreasing hypertrophic signaling downstream of ET-1 in cardiomyocytes were suggested by findings made previously by the authors that antihypertrophic effects in isolated rat hearts could be achieved by inhibition of calcineurin activity, with resulting reduction of the nuclear translocation of NF-AT, a pro-hypertrophic transcription factor (Haines et al. 2000). These experiments did not include the role of β-E in signaling; however, it was described in a 2015 report that a natural compound called tanshinone IIA inhibited estrogen receptor-mediated cardiomyocyte hypertrophy through enhancement of Akt phosphorylation. This resulted in reduced expression of calcineurin and subsequent suppression of translocation of NF-AT, with consequent inhibition of hypertrophic signaling (Weng et al. 2015). Interestingly, tanshinone has recently been observed to affect estrogen receptor activity in ways that alleviates angiotensin II-mediated cardiac hypertrophy (Chen et al. 2017). The convergence of this mechanism with aspects of cardioprotection reported by Haines et al. (2000) led to speculation that β-E, which is structurally very similar to tanshinone IIA, also offered potential for affecting these processes in ways that might ultimately prove valuable in patient treatment. It was further hypothesized that since estradiol-mediated cardioprotection through mechanisms that prominently include induction of heme oxygenase-1 (HO-1) (Zordoky and El-Kadi 2008), activity of this enzyme would likely play a role in cellular activities investigated by experiments described in the present report. It is also important to emphasize that the choice of β-E for this investigation was based on some intriguing reports, suggesting that it might prove to be a superior choice for prevention and remediation of ET-1-mediated disorders. Mounting evidence indicates that the hormone holds enormous promise in counteracting ET-1-driven deterioration of both cardiovascular and neurological functions in the aftermath of stroke (Stoop et al. 2017). Data presented in this study reveals notably that administration of the hormone following transient middle cerebral artery occlusion (tMCAO) occurring as a pathological outcome of intracerebral ET-1 injection resulted in diminished levels of degenerating neurons and lesser infarct zone magnitude, demonstrating a clear neuroprotective effect mediated by β-E. These results may demonstrate the potential for management of cardiac hypertrophy in a model that uses ET-1 treatment to induce the effects described. The study further suggests that β-E may prove useful for future clinical management of disorders in which ET-1 is a contributor to pathogenesis. This drug, a potent agonist of the nuclear steroid hormone estrogen receptor (ER), binds to both the α and β isoforms of this receptor, resulting in alteration of both gene transcription and protein expression in cells that express these receptors. The compound may also exert non-genomic effects through interaction with membrane estrogen receptors (mERs), such as estradiol-selective G protein-coupled estrogen receptor (GPER, formerly known as GPR30) (Prossnitz and Barton 2014).

ET-1 is a 21 amino acid peptide, which is a member of a family of related proteins expressed ubiquitously by all vertebrates and utilized primarily for hemodynamic regulation. Posttranslational regulation of its availability occurs through conversion of its precursor molecule, called preproendothelin-1 (PPET-1) to the bioactive form, that when expressed by vascular endothelial cells, act as a potent vasoconstrictor (Agapitov and Haynes 2002). Pathological overproduction of ET-1 results in excessive pulmonary vascular resistance, which is a major feature of pulmonary arterial hypertension (PAH). Blockade of the ET-1 receptor with the sulfonamide drug bosentan (Tracleer) ameliorates this condition by reducing pulmonary vascular resistance (Gehin et al. 2016). Cardiac hypertrophy typically occurs in response to several stressors—often associated with prior infarction, heart failure, valvular and ischemic disease, and other conditions. The primary pathologies may result in effects such as blood volume or pressure overload and diminished mass of functional contractile tissue (Haines et al. 2000). These features are well-established characteristics of the hypertrophic heart and have been known for about half a century at the time of this writing (Sandler and Dodge 1963; Hood et al. 1968). Hypertrophic growth may have evolved as an adaptive mechanism allowing for more efficient utilization of oxygen and reduced wall stress (Grossman et al. 1975). It has also been reported that both rat and human hypertrophic right ventricular tissues show upregulation of ET-1 protein levels, which might be a compensation to preserve contractility (Nagendran et al. 2013). The present investigation was structured to evaluate a general hypothesis that the prohypertrophic effects of ET-1 on both cardiomyocytes in culture and in live animals may be effectively counteracted with β-E through HO-1-associated mechanisms. One implication of this study is that use of ET-1 receptor antagonists (ERAs)—which have regulatory approval for treatment of PAH in several countries, might adversely affect right ventricular function (Nagendran et al. 2013)—hence, studies such as the present one, which provide improved insight into hypertrophic cellular processes, will enhance both safety and efficacy of various therapies for potentially pathological effects of ET-1. β-E is a potent endocrine modulator which is known to cause adverse side effects when administered exogenously (Rossouw et al. 2002). Thus, any new clinical protocols to which outcomes described by the present investigation contribute should take these properties of the hormones into account as precautions that caregivers must be aware of.

Most importantly, within the ET-1 dose range tested, this stimulant significantly increased cell size and decreased both cell viability and HO-1 protein content and activity after 48 h. Moreover, as revealed by data shown in this report, β-E at a dosage of 200 nM significantly counteracted these effects by cardiomyoblasts and counteracted ET-1-mediated HO-1 suppression. However, it is here acknowledged that effects of ET-1 and β-E with biological significance are likely to result from stimulation outside the dose ranges for both drugs—e.g., after 72 h. Nevertheless, since this investigation was configured as a preliminary study with the primary purpose of evaluating the H9c2 rat cardiomyoblast model as an investigative tool, the dose and time for cell culture treatments appear well-suited for preliminary demonstration of the cellular response trends of major interest.

In addition to PAH, inappropriately elevated ET-1 activity is known to result in a wide range of disease conditions, particularly hypertrophy-associated CVD (Li et al. 2016a). Here, we evaluate the effect of ET-1 to induce cardiac hypertrophy-associated phenotypic changes in H9c2 rat cardiomyoblast cells, an in vitro model with widely accepted reliability in cardiovascular drug discovery (Singh et al. 2015). The outcomes described here also include assessment of the capacity of intravenously administered ET-1 to affect levels of HO-1 in plasma and left ventricular heart tissue of Sprague-Dawley rats, an in vivo model, with acknowledged possible relevance to human CVD. The experiments described here further assess the capacity of the anti-inflammatory drug β-E to mitigate the effect of ET-1, yielding insight as to potential use of β-E in treatment of diseases in which ET-1 is an etiologic factor. The experimental design applied to achieve the objectives of the present study included evaluations of both cellular and systemic effects of ET-1, β-E, and HO-1 in a rat model. These processes have well-established analogs in humans (Agapitov and Haynes 2002; Gelfand et al. 1997; Czompa et al. 2014)—hence, the model used was thus considered appropriate for providing clinicians and researchers with a paradigm in which these three agents might contribute to development of future approaches to management of cardiac hypertrophy.

It is important to emphasize that the present study was not undertaken with the objective of comprehensively characterizing the full range of ET-1-induced hypertrophic effects. Such an investigation would require fairly extensive animal evaluation of each of the outcome measures shown here, which would have been beyond the scope of this effort. The experiments were designed, with a fairly narrow focus, specifically, to demonstrate the efficacy of a simple, well-established, and inexpensive in vitro model of cardiac hypertrophy as a major investigative tool for more comprehensive studies—with the corollary benefit for adding insight to effects of ET-1 and β-E on HO-1 expression, which will have direct value to both researchers and healthcare providers, who use activity of this enzyme as a lab biomarker and clinical correlate of cardiac symptoms.

## Materials and methods

### Summary of methods: rationale for major features of experimental design

The present study used H9c2 rat cardiomyoblast cells as an in vitro model based on their proven capacity to exhibit physiologic responses useful in drug discovery for cardiovascular medicine (Chen et al. 2013). The useful characteristics of these cells notably include ET-1-mediated hypertrophic effects (Li et al. 2016b), cell surface area, and viability (Hua et al. 2014)—along with outcomes noted by the authors, in which Western blot analysis was used to determine HO-1 expression by these cells (Csepanyi et al. 2015). Likewise, Sprague-Dawley rats, the animal model selected for these experiments has a well-established record in cardiovascular research, including investigations conducted by the authors which also utilize methods described here (Czompa et al. 2014). The animals were anesthetized with ketamine for reasons of compassion and to minimize confounding influence of stress. One gender (male in this case) was used to avoid potentially confounding influence of sex differences on experimental outcomes. A secondary rationale for selection of male versus female animals was to be certain that estradiol, a female sex hormone, exerted effects on male subjects—thus providing preliminary evidence that the drug holds promise for clinical treatment of ET-1-related pathologies in males—and probably not limited to female patients.

### H9c2 rat cardiomyoblast cell culture

H9c2 rat cardiomyoblast cells (American Type Culture Collection, Manassas VA, USA) were cultured in Dulbecco’s modified Eagle’s medium (DMEM) supplemented with 10% fetal bovine serum (FBS) and antibiotics (1% penicillin-streptomycin), then incubated at 37 °C in the presence of 5% CO_2_. Cardiomyoblast cells were plated onto 75-cm^2^ TPP culture dishes. Subculturing of cells was conducted serially at approximately 60–70% confluence. The subculturing protocol is briefly described as follows: the old medium is first decanted from the adherent cardiomyoblasts in each plate, then washed with phosphate-buffered saline (PBS, at pH = 7.4) and incubated in trypsin-EDTA solution for 15 min. Cells were resuspended and centrifuged for 6 min at 1100 rpm and at a temperature of 26 °C. After centrifugation, the supernatant was discarded and the pellets resuspended in fresh FBS maintenance medium. The cells were next cultured on 75-cm^2^ TPP plates at a concentration of ~ 100,000 cells/ml.

### Induction of hypertrophy in H9c2 cardiomyoblasts

The in vitro hypertrophy induction protocol for the H9c2 cardiomyoblast cell model (Watkins et al. 2010) included treatment with the hypertrophic agonist endothelin-1 (ET-1, E7764, Sigma, Germany). For these procedures, cardiomyoblasts were cultured to optimally control the hypertrophic responses of ET-1. Ethanol (E) served as the solvent in each group. Cardiomyoblasts were washed, cultured, and treated with serum-free medium and 1% of antibiotics, in maintenance DMEM, for the duration of each experiment. Cells were then incubated for 24 h at 37 °C, and cultured in the presence of 5% CO_2_. After 24 h, the cardiomyoblasts were exposed to 100, 1000, and 10,000 nM of ET-1, respectively. Cells were pretreated with 200 nM of β-E (E2758, Sigma, Schnelldorf, Germany) for 6 h before exposing to 1000 nM of ET-1.

### Cell surface area (cell size)

Morphological studies of H9c2 cardiomyoblasts to evaluate the magnitude of cell surface area (Pedram et al. 2005) were accomplished using TPP 24-well plates, in which ~ 8000 cells per well were cultured on glass coverslips. Cells were treated as described above, then observed using fluorescence microscopy. After 48 h following the hypertrophic challenge, cells were fixed for 15 min in 4% of formalin, washed three times and extracted in 0.1% of Triton-X solution for 15 min. Fluorescein isothiocyanate (FITC)-conjugated phalloidin was used to visualize cytoskeletal structures and 4′.6-diamidino-2-fenylindol (DAPI) stain for nuclear elements. The Zeiss Axio Scope A1 fluorescent microscope was used with ZEN 2012 software to acquire images and for measurements of cell area in square micrometers (μm^2^). Microscopy observations were expressed as the mean of four independent experiments, using 150 cells in each group.

### Cell viability

The viability of H9c2 cardiomyoblast cells was determined following treatment with ET-1 and β-E. Cells were seeded onto TPP 96-well microplates at an average density of ~ 2500 cells per well. Cardiomyoblasts were pretreated with β-E, and then cultured for 48 h with the hypertrophic agent ET-1. Next, 20 μl of yellow MTT (3-(4.5-dimethylthiazol-2-yl)-2.5-diphenyltetrazolium bromide) was added to cell culture medium at a volume of 5 mg/ml. This compound is reduced to purple formazan in the mitochondria of living cells, allowing their easy identification. Cells were incubated with MTT for 210 min at 37 °C, followed by removal of this staining medium and resuspension of purple crystalline marker using isopropyl alcohol. The cells were next incubated for 30 min in a thermostat at 37 °C. Absorbance was then measured at wavelengths of 570 and 690 nm, using a FLUOSTAR Optima spectrophotometer.

#### HO-1 protein expression in H9c2 rat cardiomyoblasts

H9c2 rat cardiomyoblasts, approximately 100,000 cells in each group, were treated with 1000 nM of ET-1, 200 nM of β-E, and 200 nM of β-E followed by 1000 nM of ET-1, respectively. Ethanol (E) (0.01%) served as the solvent in various groups. Following 48 h of culture, HO-1 protein levels, in drug-free control and each drug-treated group, were determined. HO-1 proteins were determined at the Food Analytical Laboratory of University of Debrecen, Hungary, using the StressXpress™ Human HO-1 ELISA Kit (Enzo Life Sciences International, Inc., PA, USA). Assays using the cultured cardiomyocyte model defined by the culture conditions are shown in legend in Fig. 3. Briefly, cells were incubated in 96-well microtiter plate, in each group, coated with an antihuman HO-1 antibody, followed by treatment with secondary/detect antibody and related reagents provided with kits. HO-1 levels were evaluated during the absorbance of the developed kit reagents at 450 nm in a Biotek ELX 808 Microplate Reader. Results were expressed in nanograms of HO-1 protein, as median values of each of four ELISA outcomes (quadruplicates) within the designated interquartile range for the Enzo kits used to terminate HO-1 assays.

### Western blots: in vitro and in vivo

Approximately 100,000 of H9c2 rat cardiomyoblasts (in 1 ml) were homogenized and lysed in 1 ml isolating buffer containing 25 mM Tris-HCl, 25 mM NaCl, 1 mM orthovanadate, 10 mM NaF, 10 mM pyrophosphate, 10 mM okadaic acid, 0.5 mM EDTA, 1 mM PMSF, and 1× protease inhibitor cocktail. Homogenates were centrifuged at 2000 rpm at 4 °C for 10 min, and the supernatants were transferred to new tubes, and further centrifuged at 10,000 rpm at 4 °C for 20 min, and the resultant supernatants were used as cytosolic extract. The protein concentration was determined by a BCA Protein Assay Kit (Thermo Scientific, Rockford, IL, USA) using bovine serum albumin (BSA) as the standard. A total of about 100 μg of protein in each sample were run on 12% acrylamide gels (Bio-Rad Laboratories, Hercules, CA, USA). Assays for both HO-1 and its reference housekeeping gene (GAPDH) were performed on the same blot. The blots were next transferred to a polyvinylidene difluoride (PVDF) membranes (Bio-Rad Laboratories, Hercules, CA, USA) for 1 h at 100 V. After blocking the membranes with 5% of non-fat dry milk in Tris-buffered saline with 0.1% Tween 20 (TBST), membranes were incubated overnight with primary antibody solution of HO-1 1/1000 (Abcam, Cambridge, UK). Antibodies to GAPDH, the housekeeping gene product used as a reference marker for assay of HO-1 protein expression, were obtained from Cell Signaling Technology, Boston, MA, USA. Subsequent to the above treatment, the membranes were washed with TBST three times and incubated with the horseradish peroxidase (HRP)-conjugated secondary antibody solution (1/3000 Cell Signaling Technology) for 1 h at room temperature. After washing, the membranes were treated/incubated with Clarity Western ECL Substrate (Bio-Rad Laboratories, USA) to visualize the bands. Following the ECL treatment, the membranes were exposed on X-ray films (Agfa, Mortsel, Belgium). The blots were then digitalized and analyzed using ImageJ program (Bak et al. 2003; Czompa et al. 2014).

For in vivo studies, following 48 h of treatments with ET-1 (1000 ng/kg), β-E (200 ng/kg), and β-E (200 ng/kg) + ET-1 (1000 ng/kg), respectively, Sprague-Dawley rats (230–290 g) were anesthetized with ketamine to avoid neuronal and hormonal stress as confounding influences in the experiments. The chest was opened and hearts were excised. Then, blood was washed out by Krebs-Henseleit buffer via the aorta. About 10 mg from left ventricular tissue was used, homogenized, and centrifuged for Western blot study. Briefly, myocardial tissue was homogenized and suspended (50 mg/ml) in sample buffer (10 mM HEPES, pH 7.3, 11.5% sucrose, 1 mM EDTA, 1 mM EGTA, 1 mM diisopropyl fluorophosphate, 0.7 mg/ml pepstatin A, 10 mg/ml leupeptin, and 2 mg/ml aprotinin). Proteins were then solubilized with Laemmli solution [9% SDS (wt/vol), 6% mercaptoethanol (vol/vol), 10% glycerol (vol/vol), and a trace amount of bromphenol blue dye in 0.196 M Tris-HCl (pH 6.7)]. Proteins (50 μg protein) were electrophoresed through 10% SDS-PAGE and transferred to Immobilon-P membranes (Millipore, Billerica, MA) using a transfer system (Bio-Rad, Hercules, CA). The blots were blocked in Tris-buffered saline/Tween-20 with 5% BSA for 1 h and incubated with antibody for recombinant rat HO-1 protein (StressGen Biotechnologies, Victoria, BC, Canada). Membranes were incubated with anti-goat IgG and were exposed to film (Amersham, UK).

### Stimulant effects on HO-1: in vivo studies

Male Sprague-Dawley rats (230–290 g) were purchased from Charles River Laboratories International, Inc. (Sulzfeld, Germany). All animals used for experiments in the present investigation received humane care in compliance with the *Guide for the Care and Use of Laboratory Animals* published by the US National Institutes of Health (NIH Publication No. 85-23, revised 1996) and approved by the local Committee of the University of Debrecen (UD), Debrecen, Hungary. UD ethical committee approval certificate number: 3/2012/DE MÁB.

Rats were fed with commercial food pellets and water ad libitum and treated intravenously with 1000 ng/kg of ET-1, 200 ng/kg of β-E, and 200 ng/kg of β-E followed by 1000 ng/kg of ET-1, respectively. Ethanol (E) (0.01%) served as the solvent in various groups, respectively. After 48 h following the treatment, about 1 ml of venous blood was taken from untreated and drug-treated rats, centrifuged, and then plasma HO-1 protein levels were determined using the StressXpress™Human HO-1 ELISA Kit (Enzo Life Sciences International, Inc., PA, USA). Plasma was incubated in 96-well microtiter plate coated with antirat HO-1 antibody, followed by treatment with secondary/detect antibody and related reagents provided with kits. Plasma HO-1 levels were evaluated during the absorbance of the developed kit reagents at 450 nm in a Biotek ELX 808 Microplate Reader. Results are calculated in nanograms of HO-1 protein per milliliter of plasma as median values of each of four ELISA outcomes (quadruplicates, *n* = 8 measurements) within the designated interquartile range for the Enzo kits used to accomplish these HO-1 assays.

### Statistical analyses

Results of cell surface area (cell size), cell viability, and HO-1 protein expression were compared between groups by using analysis of variance (ANOVA) followed by Dunnetts’s test (Wallenstein et al. 1980). The results obtained in each drug-treated group were compared to the values obtained in the drug-free control (C) or the 1000 nM of ET-1-treated group, respectively. The values obtained were expressed as the means ± standard error of the means (SEM) and were considered significantly different at *p* < 0.05.

## Results

### Cell surface area changes in response to hypertrophic stimulus

**Fig. 1:**
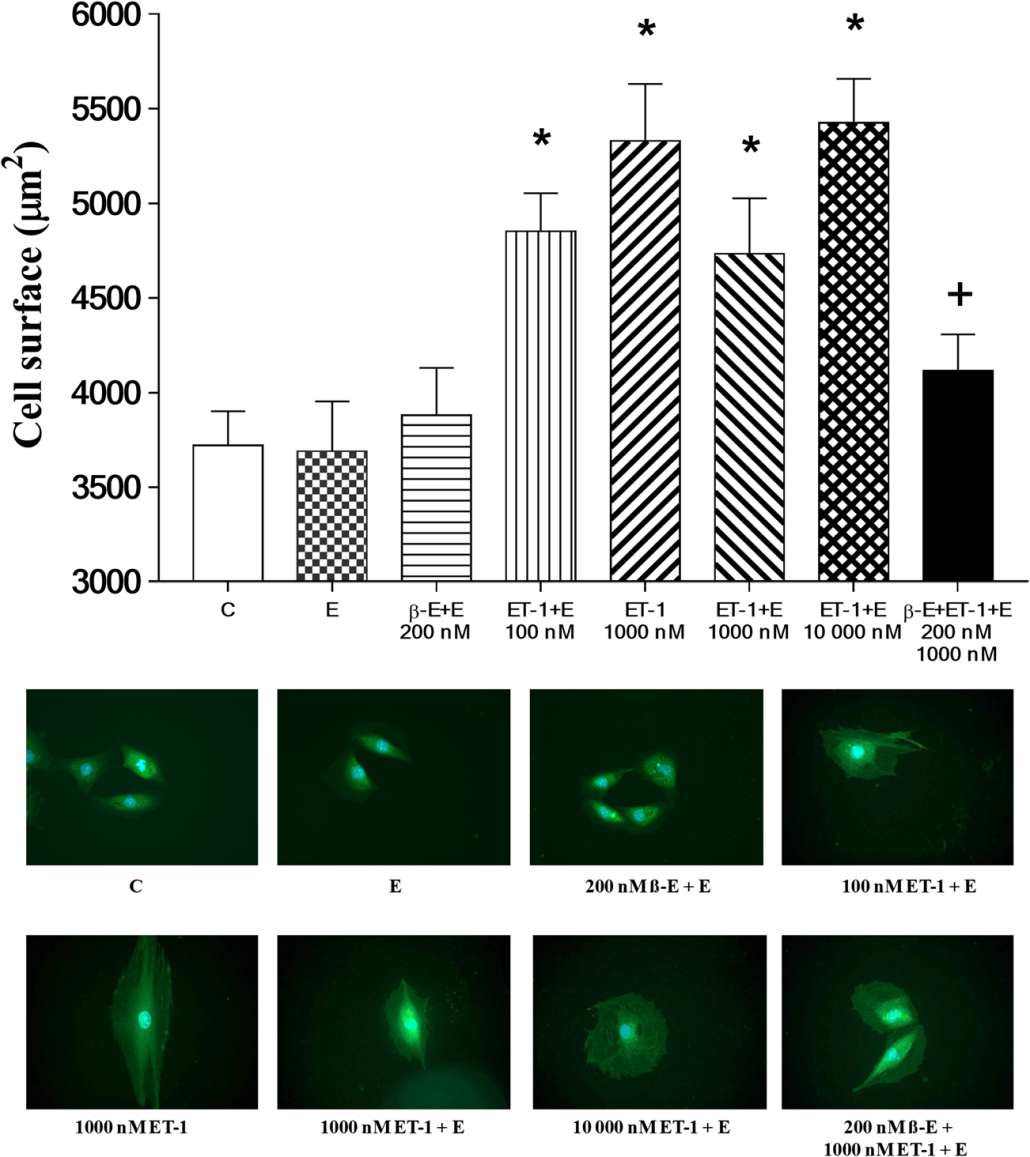
Cell surface area magnitude changes in response to hypertrophic challenge. The surface area of H9c2 rat cardiomyoblasts was measured in cells cultured under the following conditions: unsupplemented cell culture medium (control, C), cell culture medium containing 0.01% of ethanol (E), 200 nM of β-estradiol (β-E) in medium containing 0.01% of ethanol (200 nM β-E + E), 100 nM of endothelin-1 (100 nM ET-1) in medium containing 0.01% of ethanol (100 nM ET-1 + E), 1000 nM of ET-1 in unsupplemented cell culture medium (1000 nM ET-1), 1000 nM of ET-1 in medium containing 0.01% of ethanol (1000 nM ET-1 + E), 10,000 nM of ET-1 in medium containing 0.01% ethanol (10,000 nM ET-1 + E), and 200 nM of β-E and 1000 nM of ET-1 in medium containing 0.01% of ethanol (200 nM β-E + 1000 nM ET-1 + E). Outcomes are expressed in square micrometers of cell surface area (μm^2^) ± SEM. *n* = 200 cells in each group. *p < 0.05 with respect to comparisons made to the drug-free control culture (C) value, and ^**+**^*p* < 0.05 compared to the average percent cell viability evaluated in the 1000 nM of ET-1 + E group

The histogram in the upper panel of Fig. 1 demonstrates that H9c2 cells treated with 100, 1000, and 10,000 nM of ET-1 exhibited significant increases in the average cell surface areas (representing cell size), in the presence or absence of ethanol (E) in comparison with the drug-free control value (C) (*p* < 0.05). Ethanol (E) concentration was constant in each of the experimental conditions described. It was further noted that treatment with ethanol or β-E did not cause significant changes in cell size and other outcomes measured when results were compared with data obtained from drug-free control group (C). Continuation of these studies, in which cells were treated with 200 nM of β-E, followed by stimulation with 1000 nM of ET-1, revealed that β-E-mediated significant suppression of cell surface area increase—induced by 1000 nM ET-1. The lower panel of Fig. 1 shows representative size comparisons of cells in the drug-free control (C) and β-E- and ET-1-treated groups. These photographs demonstrate that cells treated with 100, 1000, and 10,000 nM of ET-1, respectively, resulted in cell volume increases, with β-E-mediated suppression of this hypertrophy-associated effect. These results demonstrate that coadministration of 1000 nM of ET-1 and 200 nM of β-E resulted in a reduction in cell size in comparison to cells cultured with 1000 nM of ET-1.

### Stimulant effects on cell viability

**Fig. 2:**
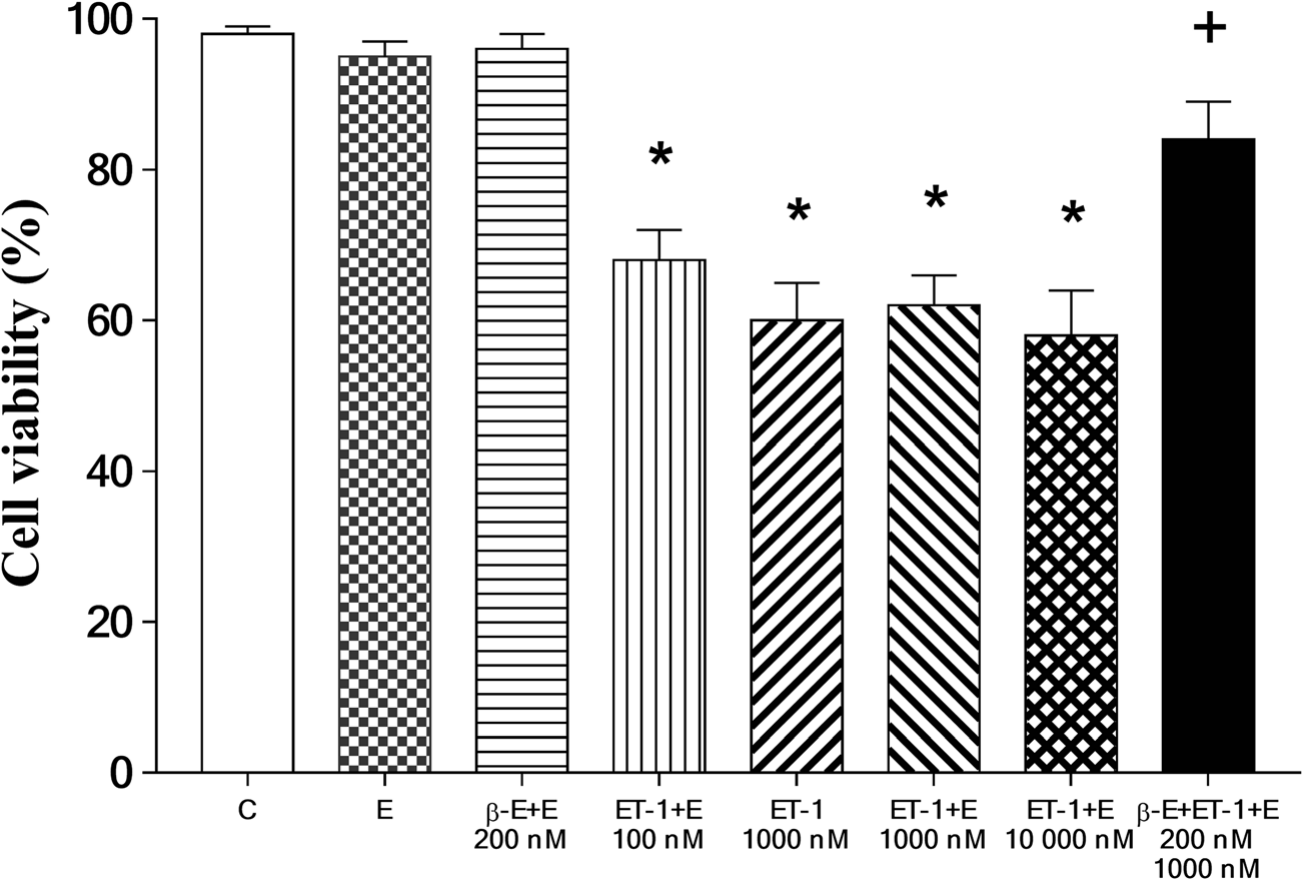
Effect of hypertrophic challenge on cell viability in cell cultures. H9c2 cardiomyoblast viability was assessed using the MTT assay in the following groups: unsupplemented cell culture medium (control, C), cell culture medium containing 0.01% of ethanol (E), 200 nM of ß-estradiol (β-E) in medium containing 0.01% of ethanol (200 nM β-E + E), 100 nM of endothelin-1 (ET-1) in medium containing 0.01% ethanol (100 nM ET-1 + E), 1000 nM of ET-1 in unsupplemented cell culture medium (1000 nM ET-1), 1000 nM of ET-1 in medium containing 0.01% of ethanol (1000 nM ET-1 + E), 10,000 nM of ET-1 in medium containing 0.01% ethanol (10,000 ET-1 + E), and 200 nM of β-E and 1000 nM of ET-1 in medium containing 0.01% of ethanol (200 nM β-E + 1000 nM ET-1 + E). *n* = 150 in each group. *p < 0.05, comparisons were made to the drug-free control (C) value; ^+^*p* < 0.05, comparisons were made to the ET-1 (1000 nM ET-1) and all ET-1 + E-treated groups

As shown in Fig. 2, relative to viability of cells cultured in unsupplemented medium, cardiomyocytes grown in 0.01% of ethanol and 200 nM of β-E, were not significantly affected. Conversely, it was observed that treatment with ET-1 significantly reduced viability of cells administered 100–10,000 nM of this stimulant. It was further noted that pretreatment of cells with 200 nM of β-E significantly protected against ET-1-mediated cell death in comparison with the entire dose range evaluated.

### Stimulant effects on expression of HO-1: in vitro studies

**Fig. 3:**
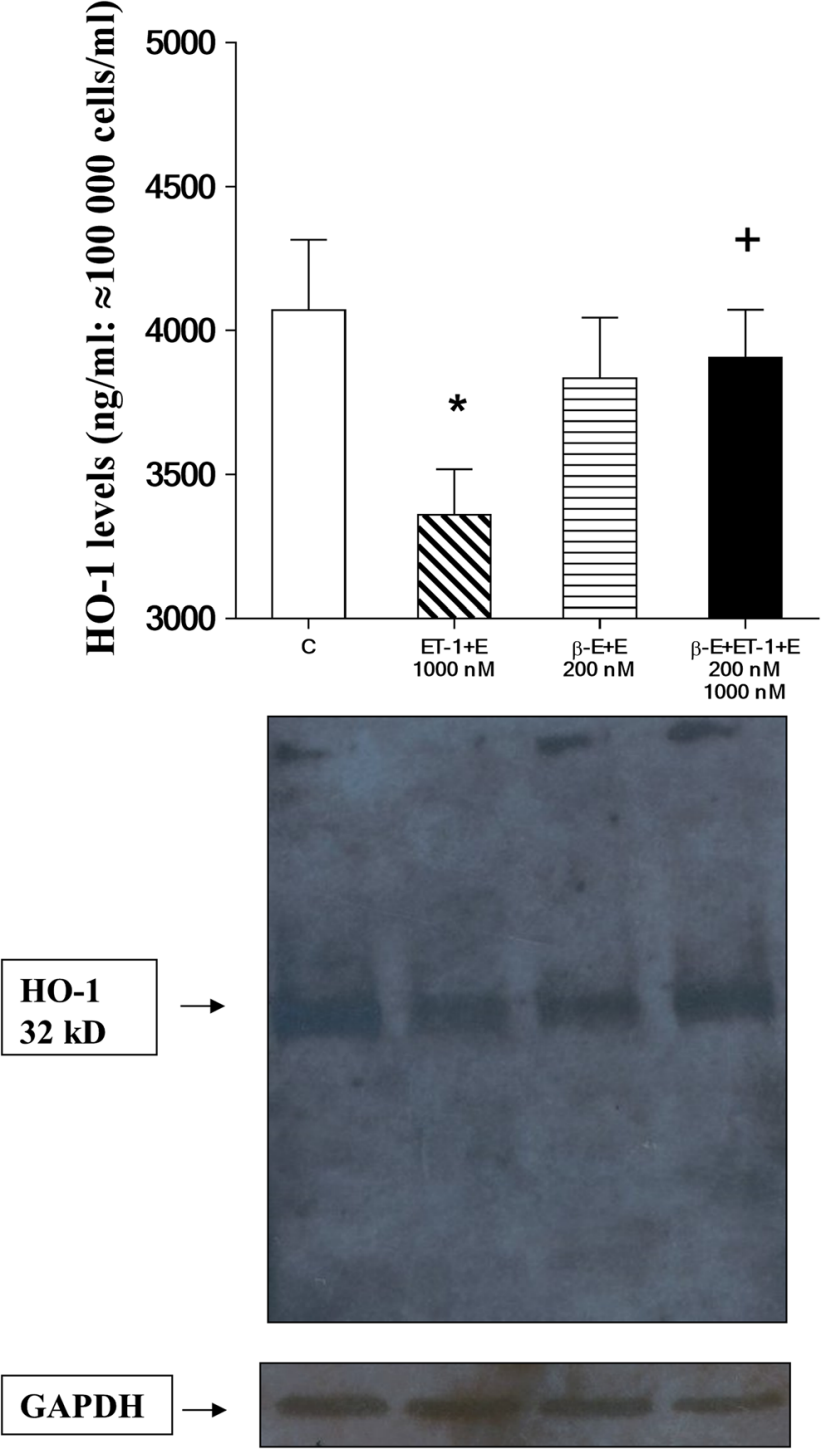
ELISA analysis of heme oxygenase-1 (HO-1) protein levels and Western blots in in vitro cell culture. H9c2 rat cardiomyoblast expression of HO-1 protein was assessed in cells cultured under the following conditions: unsupplemented cell culture medium (control, C), 1000 nM ET-1 in medium containing 0.01% of ethanol (1000 nM ET-1 + E), 200 nM β-estradiol in medium containing 0.01% of ethanol (200 nM β-E + E), and 200 nM of β-E and 1000 nM of ET-1 in medium containing 0.01% ethanol (200 nM β-E + 1000 nM ET-1 + E). ELISA analysis results shown in the upper panel for HO-1 protein levels. Results are shown as average HO-1 levels. *n* = 8 in each group. Western blot representative bands corresponding with the bars (figure upper part), shown in the middle panel, was obtained from cytosolic extracts of cardiomyoblast lysates. The expression of glyceraldehyde 3-phosphate dehydrogenase (GAPDH) was measured as a reference housekeeping protein (lower panel). *p < 0.05, comparisons were made to the drug-free control (C) value; ^+^*p* < 0.05, comparisons were made to the ET-1 + E (1,000 nM)-treated group

Expression of HO-1 protein by H9c2 rat cardiomyoblasts in response to indicated stimulation conditions for 48 h is shown in Fig. 3. The histogram in the upper part of the Fig. 3 demonstrates that cells cultured with 1000 nM of ET-1 in medium containing 0.01% of ethanol (ET-1 + E, 1000 nM), produced significantly less HO-1 than those incubated in unsupplemented drug-free medium (C) (*P* < 0.05). The effect of β-E on cardiomyocyte expression of the enzyme, assessed by treatment with 200 nM of β-E with 0.01% of ethanol included as a solvent for the drug (β-E of 200 nM + E), revealed no significant alteration by these stimulation conditions relative to cells grown in unsupplemented control (C) medium. β-E at a dosage of 200 nM was observed to result in significantly restored HO-1 expression by cardiomyoblasts treated with 1000 nM of ET-1 (β-E of 200 nM + ET-1 of 1000 nM + E) relative to cells cultured with 1000 nM of ET-1 without β-E (ET-1 of 1000 nm + E) (*P* < 0.05). Results of Western blot analysis shown in the lower frame of Fig. 3 revealed bands corresponding to HO-1 (MW = 32 kDa) and GAPDH (MW = 37 kDa), detection antibodies for which were obtained from Cell Signaling Technology (Boston, MA, USA). Subjective visual comparison of relative band intensity for HO-1 protein parallels outcome shown in Fig. 3, demonstrating three major results: first, that ET-1 treatment of cardiomyoblasts suppresses HO-1 protein levels and expression; secondly, that β-E alone has no significant effect on production of the enzyme; and thirdly, that β-E is capable of restoring a significant fraction of HO-1 expression by cells, in which it is suppressed by ET-1 (1000 nM) treatment.

### Stimulant effects on HO-1: in vivo studies

**Fig. 4:**
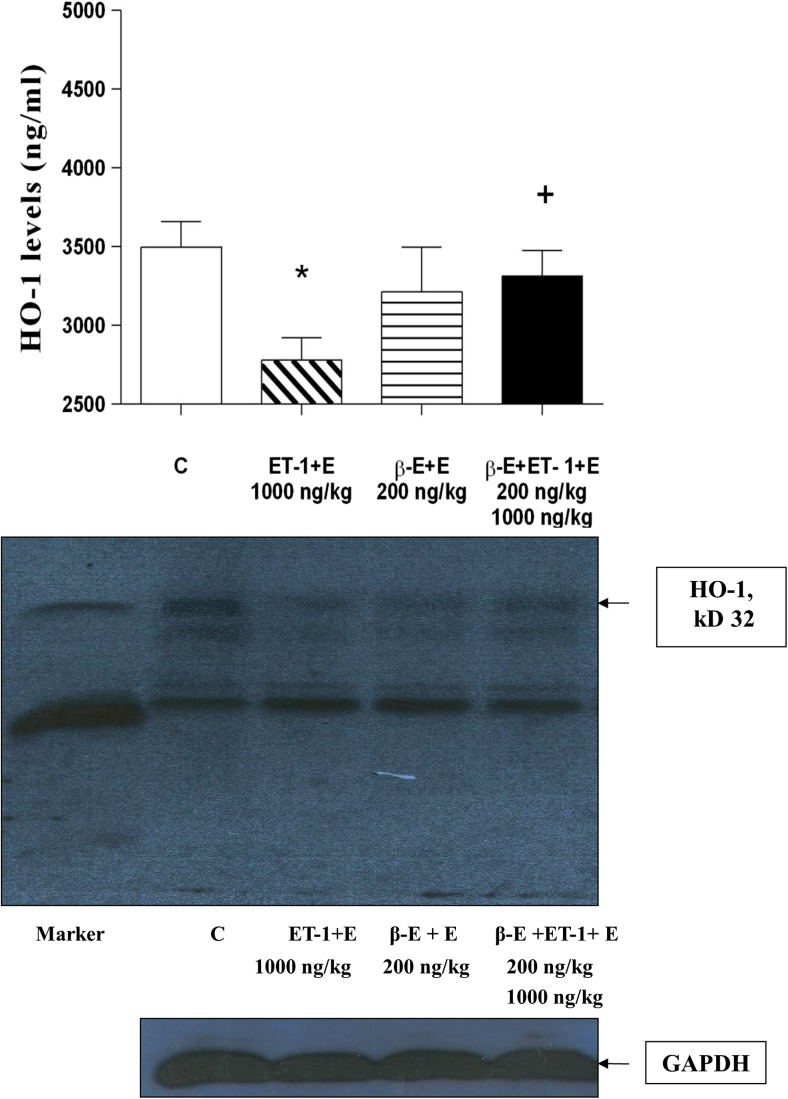
ELISA analysis of heme oxygenase-1 (HO-1) protein levels in plasma and Western blots in myocardial tissue. HO-1 levels in plasma protein (upper panel) and expression of HO-1 protein (middle panel) in left ventricular tissue assessed under the following conditions: rats were treated with (i) 1000 ng/kg of endothelin-1 (1000 ng/kg ET-1 + E), (ii) 200 ng/kg of β-estradiol (200 ng/kg β-E + E), and (iii) 200 ng/kg of β-E and 1000 ng/kg of ET-1 (200 ng/kg β-E + 1000 ng/kg ET-1 + E), respectively. Ethanol (E) was the solvent at a final concentration of 0.01% in each sample. Results are shown as average of HO-1 plasma protein levels (upper panel). *n* = 8 (quadreduplicated) in each group. Western blot representative bands corresponding with the bars (upper part), shown in the middle panel, was obtained from lysates of left ventricular tissue. The expression of glyceraldehyde 3-phosphate dehydrogenase (GAPDH) shown as a reference housekeeping protein (lower panel). *p < 0.05, comparisons were made to the drug-free control (C) value; ^+^*p* < 0.05, comparisons were made to the ET-1 (1000 ng/kg)-treated group

Plasma HO-1 protein levels in peripheral plasma collected 48 h following intravenous treatment of rats with endothelin-1 and β-E are shown in the upper panel of Fig. 4. Data shown by histogram displays in the upper panel of the Fig. 4 demonstrate that relative to serum of untreated animals, plasma from rats treated with 1000 ng/kg of ET-1 contained significantly lower amounts of HO-1 protein (*P* < 0.05). Whereas plasma HO-1 levels in animals coadministered 1000 ng/kg of ET-1 with 200 ng/kg of β-E, exhibited HO-1 content with no statistically significant difference from levels of the enzyme in untreated control (C) rats. Outcomes of the evaluation of β-E effect on plasma HO-1 levels also shown in the upper panel of Fig. 4 reveal that animals receiving 200 ng/kg of this drug exhibit HO-1 content of plasma not significantly different from plasma of rats receiving 1000 ng/kg of ET-1 and 200 ng/kg β-E, or untreated control animals. Results of representative Western blots conducted on myocardial tissue collected 48 h following ET-1 and β-E administration are shown in the middle panel of Fig. 4. These results show bands corresponding to HO-1 (MW = 32 kDa) and GAPDH (MW = 37 kDa) in the lower panel of Fig. 4. Visual comparison of relative band intensity for HO-1 protein parallels outcomes of assays for HO-1 shown in the upper panel of the Fig. 4. Specifically, data in Fig. 4 demonstrates that intravenous ET-1 treatment reduces HO-1 expression in both plasma and myocardial tissue. Results shown in Fig. 4 also demonstrate that β-E alone has no significant effect on HO-1 expression in plasma or myocardial tissue. Results shown in the middle panel of Fig. 4 represent outcomes of an experiment which was conducted to generate a representative qualitative picture of response of the model to stimulation—without statistical workup (i.e., non-quantitative study). These in vivo outcomes were intended to provide a rough single visual indicator of HO-1 expression response for the purpose of extending this and future study design in ways that will allow improved use of the animal model in HO-1 expression investigations. It is important to note that the effect of ET-1 or its release by tissue is very different in cell models, isolated organ models, or intact animals (Szolcsanyi et al. 2001; Lekli et al. 2008). Finally, these outcomes reveal that β-E is capable of preserving production of the enzyme systemically and by heart tissue.

## Discussion

Emerging trends in investigation of the biological properties of ET-1 and relevance of this vasoactive protein to cardiopulmonary disorders are driven substantially by recognition that this molecule is a critical factor in pathogenesis of diseases such as pulmonary arterial hypertension (PAH) (Kylhammar and Radegran 2016). Etiologic contributors to these diseases prominently include chronic exposure to aerial pollutants, in particular oxides of nitrogen and sulfur and partly combusted fuels (Cheng et al. 2015; Velicka et al. 2015; Ghanbari Ghozikali et al. 2016). Public health policies in nations with severe air pollution problems, particularly the People’s Republic Of China, have responded to the burden placed on their medical care facilities by prioritizing support for research into ET-1 and other biomolecules that affect respiratory and cardiovascular function (Pan et al. 2010; Luo et al. 2012). The present study evaluates strategies for affecting disease-associated ET-1 effects using the H9c2 rat cardiomyoblast model, which is widely used for investigation of cellular aspects of cardiovascular pathologies. This in vitro system is here utilized for evaluation of the ability of β-E to counteract effects of ET-1, which are features of ET-1-mediated diseases, including cardiac hypertrophy and pulmonary arterial hypertension. The results demonstrate that ET-1 stimulates changes in H9c2 cardiomyoblast cells that are also observed in cardiac hypertrophy, including increases in cell size (Porchia et al. 2008) and decreased viability (Xu et al. 2010) and reduced expression of HO-1 protein (Liou et al. 2015). The outcome of studies conducted to demonstrate ET-1-mediated changes in cell size, shown in Fig. 1, clearly indicates that major effects of this stimulation is observed in a dose range from 0 to 10,000 nM of ET-1. Outcomes in this interval represent a plateau value for cell size increase in this model. The absence of significant cell size increase in response to presence of 0.01% ethanol in culture media indicates its value for use as a solvent for β-E. Moreover, since 200 nM of β-E did not independently induce significant H9c2 cell size increase, this drug was considered appropriate for evaluation of ET-1 effects. The capacity of 200 nM of β-E to significantly attenuate 1000 nM of ET-1-mediated cell size increase demonstrates the utility of this drug in counteracting this feature of pathological processes in which ET-1 is a key factor.

The results of representative H9c2 cardiomyoblast cell fluorescent microscope images revealed increases of cell sizes under each ET-1 stimulation condition, occurring in parallel to ET-1-mediated induction of cell size increases. Although mechanistic studies were not conducted to determine molecular mechanisms of ET-1-induced increased cardiomyocyte size, previous work by other investigators demonstrated that the protein simulates high levels of activity by mammalian target of rapamycin (mTOR), a serine/threonine protein kinase which is a central regulator of cellular metabolism, growth, and survival in response to stressors, nutrients, growth factors, and hormones—moreover, ET-1-induced increases in mTOR activity, acts on a feline model of adult cardiac muscle cells to stimulate phosphorylation signaling which contributes to hypertrophic effects (Moschella et al. 2007). This effect is probably an element of mTOR-dependent processes promoting size increases of the kind observed in H9c2 cells described in the present study. Interestingly, similar mTOR-dependent mechanisms contribute to geroconversion of human cells to pro-inflammatory senescent forms—an underlying feature of physical deterioration occurring as a main aspect of the aging process (Haines et al. 2013; Leontieva et al. 2015). Overactivity of this enzyme often arrests cell division but allows accumulation of protein precursors to cell structural elements, resulting in increased cell size and disruption of normal physiologic functions, along with high expression of inflammatory mediators, which in turn cause dysregulation of tissue homeostasis—and a wide range of pathological effects (Haines et al. 2013; Leontieva et al. 2015).

The effects of ET-1 challenge on H9c2 viability were assessed using the colorimetric MTT assay as described. Outcomes of these experiments are shown in Fig. 2. Relative to viability of cells grown in unsupplemented control media, the significant decrease in H9c2 viability observed in cells treated with 100–10,000 nM of ET-1 was expected based on previous studies (Funayama et al. 2013). The mechanism by which ET-1 decreases viability of cardiomyocytes also appears to involve disruption of normal protein metabolism (Funayama et al. 2013). Dose-response evaluations were conducted for the effect of ET-1 on cells at 0, 100, 1000, and 10,000 nM (Figs. 1 and 2). Based on the outcomes of ET-1 alone, the experiments were designed to evaluate the effect of β-E at the stimulation levels shown by ET-1, on the assumption that below the dose of 100 nm of ET-1, β-E effects would not have produced significant changes in comparison with the drug-free control (C) value in our in vitro model system. Western blot data outcomes shown in Figs. 3 and 4 are most clearly interpreted in the context of results shown in the previous figures. Here again, it is important to note that ethanol at the concentration used failed to significantly influence any of the outcome variables measured; moreover, β-E alone likewise did not cause significant alterations in any of the effects measured. It is nevertheless important to note that 200 nM of β-E interacted with cultured cells subjected to 1000 nM of ET-1 to counteract hypertrophic effects of the molecule as shown in Figs. 1, 2, and 3. As part of the experimental design, the authors utilized these outcomes as a rough guide to establish the in vivo experiments in HO-1 protein expression by live rats treated with ET-1 (Fig. 4). This experimental strategy is nevertheless subject to an important caveat: specifically, the Western blot assays both for cultured cells (Fig. 3) and for live animals were configured as qualitative evaluations of ET-1-mediated induction of HO-1. Here, the authors acknowledge that the data shown in Figs. 3 and 4 absolutely do not demonstrate definitive and precisely reproducible correlations between ET-1 stimulation and hypertrophic effects. Instead, the outcomes provide readers who may consider using this study as a starting point for more comprehensive analysis of approaches to counteracting pathologies, in which ET-1 plays a role, particularly in development of safety and efficacy studies described in the introductory section of the present paper.

The results of the present study provide improved insight into specific effect of ET-1 on cells of the heart and the ability of β-E to remediate them. The findings described here will allow improved focus of experimental design for ongoing basic scientific investigations and human clinical evaluations of β-E therapy of disorders, in which ET-1 is a key contributor. The effects of ET-1 on HO-1 production or expression are particularly intriguing in the context of β-E use in patient care. β-E is an estrogen sex hormone, important in the regulation of the estrous and female menstrual cycles. It thus has significant adverse effects, including increased triglycerides, which exacerbates cardiovascular disorders (Gelfand et al. 1997). For this reason, naturally occurring, generally regarded as safe (GRAS) formulations which interact additively or synergistically with β-E to decrease its effective dosage, are of potentially enormous clinical value. Recently, HO-1 inducers have been recognized as being valuable for treatment of cardiovascular, lung, neurological, and kidney disorders (Haines et al. 2012), along with osteoarthritis as described above (Mahmoud et al. 2015).

Results of in vitro studies described above were paralleled by outcomes of experiments using a Sprague-Dawley in vivo rat model, in which peripheral blood plasma and left ventricular heart tissue animals treated intravenously with ET-1 and β-E, were assessed for expression of HO-1 protein. These data, shown in Fig. 4, reveal that relative to drug free animals peripheral plasma and cardiac muscle HO-1 content of ET-1-treated rats is significantly reduced. This result is consistent with findings made by similar analyses of cultured cardiomyocyte response to ET-1 stimulation, an indicator of correlation, expected between potentially pathological increases in ET-1 and diminished protective capacity of HO-1. Specifically, these outcomes provide additional evidence that capacity of the enzyme to counteract deleterious effects of aberrantly increased ET-1 levels in cardiovascular syndromes is impaired by influences that promote high ET-1 expression, systemically and in heart muscle tissue (Bak et al. 2003; Czompa et al. 2014). The findings are also significant for potential use of β-E as a primary therapeutic for disorders involving elevated ET-1 expression. Here, the investigators demonstrate that rats treated independently with the drug did not significantly affect HO-1 expression in blood or heart tissue in any adverse ways and restored HO-1 production to into the range of that measured in untreated control animals when given to ET-1-treated rats.

Although not specifically studied in the present investigation, it is of interest to note the findings of Csonka et al. (1999), Szabo et al. (2004), Bak et al. (2006). In their studies, authors of the abovementioned publications demonstrated a close correlation between hypertrophy-associated cellular effects and HO-1 expression and/or enzyme activities by various interventions under in vitro and ex vivo conditions in various tissues.

The results shown here demonstrate that ET-1 suppresses expression of HO-1 in H9c2 cardiomyoblast cells, thereby diminishing the cardioprotective effect of this enzyme. This result is consistent with previous studies by authors of the present report, revealing that phytochemical HO-1 inducers, typically with low or negligible occurrence of adverse side effects, are potently cardioprotective (Juhasz et al. 2013; Czompa et al. 2014; Csiki et al. 2015). These outcomes also demonstrate that HO-1 inducers offer corollary application for prevention and treatment of other severe chronic diseases, including eye disease (Szabo et al. 2004; Varga et al. 2013), type 2 diabetes (Mahmoud et al. 2013), rheumatoid arthritis (Mahmoud et al. 2014), and osteoarthritis (Mahmoud et al. 2015). These experiments did not include β-E commonalities in signaling affected by this compound as described in a 2015 report, and it was presented that a natural compound called tanshinone IIA inhibited estrogen receptor-mediated cardiomyocyte hypertrophy through enhancement of Akt phosphorylation, resulting in reduced expression of calcineurin and subsequent suppression of translocation of NF-AT, with consequent inhibition of hypertrophic signaling (Weng et al. 2015). Interestingly, tanshinone has recently been observed to affect estrogen receptor activity in ways that alleviates angiotensin II-mediated cardiac hypertrophy (Chen et al. 2017). The convergence of this mechanism with aspects of cardioprotection reported by Haines et al. (2000) led to speculation that β-E, which is structurally very similar to tanshinone IIA, also offered potential for affecting these processes in ways that might ultimately prove valuable in patient treatment.

## Conclusions

Two major findings emerging from analysis of data produced by the present study are (i) that ET-1 exerts prohypertrophic effects in the rat heart and reduces the expression of the cardioprotective enzyme HO-1, and (ii) that β-E attenuates these ET-1 effects. These effects have been demonstrated both in vitro and qualitatively in an in vivo model. The results of the present study thus open a line of drug discovery, in which the benefit of β-E may be greatly augmented by coadministration of phytochemical HO-1 inducers. The precise mechanism by which β-E achieves this protective effect via the stimulation of HO-1 remains to be resolved. Finally, results of the present investigation may underscore the value of therapeutic inducers of HO-1 in both prevention and treatment of a wide range of serious chronic disease. Particularly exciting is a recent demonstration that “non-drug” methods for amplification of this enzyme may be used to precisely target root pathomechanisms of chronic inflammatory disease, with dramatic improvement of prognoses of human patients (Haines et al. 2012).
